# Pre-cooling by hands and feet water immersion reduces heat strain while wearing protective clothing

**DOI:** 10.1186/2046-7648-4-S1-A106

**Published:** 2015-09-14

**Authors:** Ken Tokizawa, Tatsuo Oka, Akinori Yasuda, Tetsuo Tai, Son Suyoung, Jun Wada, Hirofumi Ida

**Affiliations:** 1 National Institute of Occupational Safety and Health, Kawasaki, Japan; 2 Tokyo Electric Power Company, Yokohama, Japan

## Introduction

Pre-cooling (i.e., removal of heat from the body immediately prior to exercise) is a popular strategy for improving exercise performance in hot conditions. Whole body immersion in water is the procedure most commonly used to pre-cool in sports activities. However, the supply of a large volume of water and ice in all occupational settings is not always possible, or practical. In the present study, we examined the effectiveness of hands and feet water immersion and cooling vest as practical pre-cooling method on heat strain while wearing protective clothing.

## Methods

Nine males engaged in 60 min of walking at a moderate speed (2.5 km.h^-1^) in a hot environment (37 °C, 50 % rh). Before walking, they immersed hands and feet in mild cold water (18 °C) or temperate water (28 °C) and wore a cool-vest (Phase Change Material: melting temperature 28 °C, 1680 g, 276 kJ, start from 20 °C) for 30 min. The water was wiped off and the vest was taken off, then they wore protective clothing and a full-face gas mask.

## Results

Rectal temperature (T_re_) at the end of walking was lower in the temperate- and mild cold-water cooling trials than in the control trial (without the pre-cooling) (shown in the figure, p < 0.05). Mean (SD) T_re _during walking increased by 1.1 (0.3 °C) in the control trial. The pre-cooling in the mild cold-water inhibited the increase (0.8 (0.2 °C), p < 0.05), but that in the temperate-cold water did not (0.9 (0.3 °C)). In addition, sweat rate, thermal unpleasantness, physical and psychological fatigues were lower in the pre-cooling trials than in the control trial (p < 0.05). In the mild cold-water cooling trial, the changes in heart rate, thermal sensation, and damp sensation were also attenuated (p < 0.05).

## Discussion

We additionally examined that the effect of with or without cooling vest on heat strain in the mild cold-water cooling trial. Because no significant difference was observed, hands and feet water immersion could be mainly effective on alleviating heat strain. The extremities are frequently targeted for cooling [1], as they are usually easy to access and highly vascularized [2]. This study is the first to show the pre-cooling effect of hands and feet on heat strain, whereas previous studies used the cooling procedure during and after exercise [1]. The mitigation effects in cooling at 28 °C were closer to those in cooling at 18 °C, which may be due to a less powerful peripheral vasoconstrictor response [3].

## Conclusion

Hands and feet water immersion could be an alternative pre-cooling method alleviating heat strain. Although colder water induces more physiological and psychological benefits for heat strain, using tap water (moderate) can also yield lower heat strain for a certain level.

**Figure 1:**
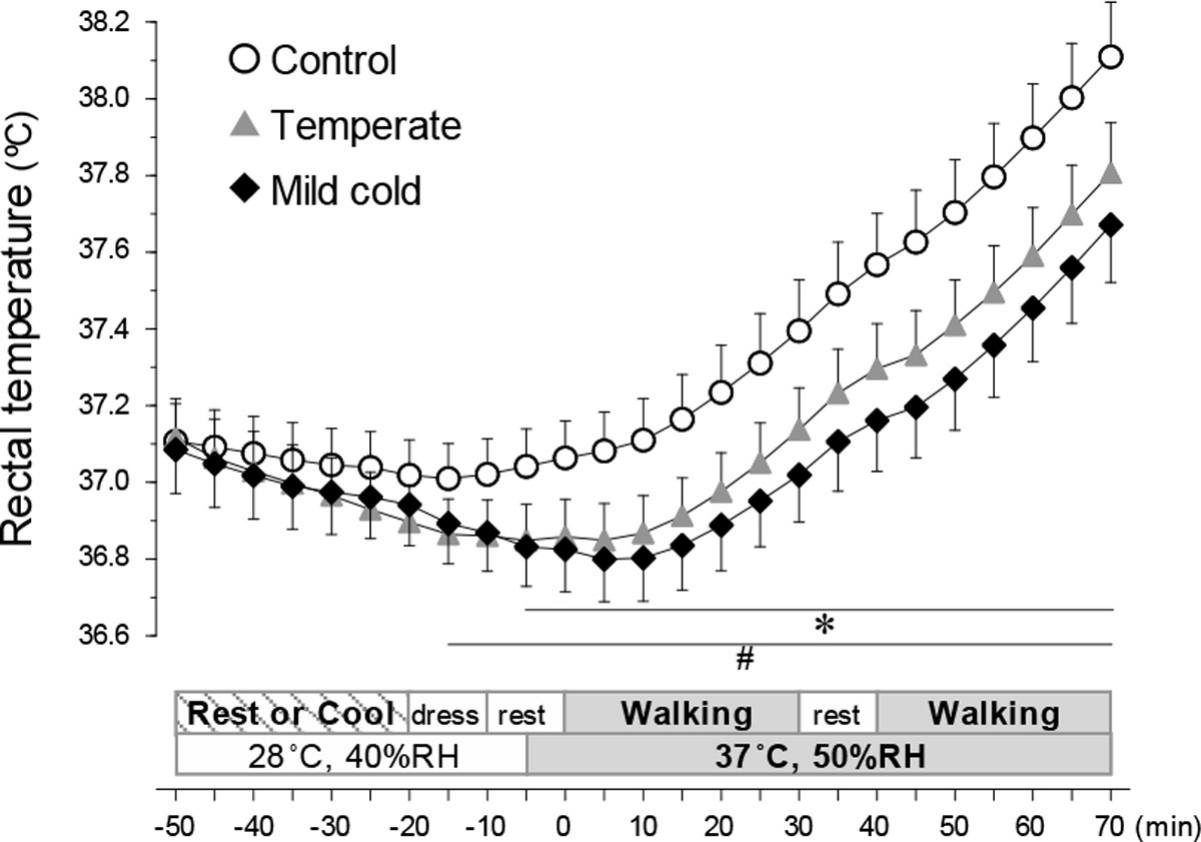
*Different (p < 0.05) between the control and mild cold-water cooling trials; #different (p < 0.05) between the control and temperate-water cooling trials.
